# Association of Wilms tumor-1 protein in urinary exosomes with kidney injury: a population-based cross-sectional study

**DOI:** 10.3389/fmed.2023.1220309

**Published:** 2023-09-18

**Authors:** Sukhanshi Khandpur, Medha Srivastava, Rajni Sharma, Shafaque Asif, Dharmendra S. Bhadauria, Prabhaker Mishra, Anil J. Purty, Swasti Tiwari

**Affiliations:** ^1^Department of Molecular Medicine and Biotechnology, Sanjay Gandhi Postgraduate Institute of Medical Sciences, Lucknow, India; ^2^Department of Nephrology, Sanjay Gandhi Postgraduate Institute of Medical Sciences, Lucknow, India; ^3^Department of Biostatistics and Health Informatics, Sanjay Gandhi Postgraduate Institute of Medical Sciences, Lucknow, India; ^4^Department of Community Medicine, Pondicherry Institute of Medical Sciences (A Unit of Madras Medical Mission), Puducherry, India

**Keywords:** non-invasive, chronic kidney disease, KIM-1, NGAL, exosomes, uE-WT1

## Abstract

**Objective:**

Loss of Wilms tumor-1 (WT1) protein, a podocytopathy marker, through urine exosome (uE), could be an early indication of kidney injury. We examined WT1 in uE (uE-WT1), along with other urine markers of glomerular and kidney tubule injury, in individuals without chronic kidney disease (CKD).

**Methodology:**

The cross-sectional study included individuals who reported having no evidence of chronic kidney disease (CKD). Albumin-to-creatinine ratio (ACR) and estimated glomerular filtration rate (eGFR) were used to assess kidney function. eGFR was calculated using the 2009 CKD-EPI (CKD-Epidemiological) equation. WT1 was analyzed in uE from humans and Wistar rats (before and after the 9th week of diabetes, *n* = 20). uE-WT1, urinary neutrophil gelatinase-associated lipocalin (NGAL), and kidney injury molecule-1 (KIM-1) were estimated using ELISA. The Kruskal-Wallis H test, Mann-Whitney U test, and stepwise multivariable linear regression were performed.

**Results:**

Urine NGAL and ACR increase with uE-WT1 quartiles (*n* = 146/quarter). Similarly, uE-WT1, KIM-1, and NGAL were positively associated with ACR. Furthermore, KIM-1, NGAL, and uE-WT1 correlated with ACR. uE-WT1 outperformed KMI-1 and NGAL to explain ACR variability (25% vs. 6% or 9%, respectively). Kidney injury in streptozotocin-induced diabetic rats was associated with a significant rise in uE-WT1. Moreover, the findings were confirmed by the histopathology of kidney tissues from rats.

**Conclusion:**

uE-WT1 was strongly associated with kidney function in rats. In individuals without CKD, uE-WT1 outperformed NGAL as a determinant of differences in ACR.

## Introduction

Chronic kidney disease (CKD) is recognized as an epidemic and a major public health problem, affecting more than 10% of the adult population. CKD accounts for ~1.2 million deaths per year (1, 2). Glomerular and kidney tubule injuries are important risks for CKD genesis (3). The dysfunction of podocytes, a significant component of the glomerular filtration barrier, is a common feature of primary and secondary glomerular disease (4–6). Although podocytes adapt to cellular stress and maintain kidney homeostasis, their irreversible loss under various pathological conditions rapidly progresses to glomerular sclerosis, leading to end-stage renal disease. Podocyte dysfunction commonly manifests as proteinuria and albuminuria (urine albumin-to-creatinine ratio, ACR≥ 30 mg/g). Besides, tubular damage can also cause albuminuria due to decreased reabsorption in the proximal tubule (7). The tubular proteins, including neutrophil gelatinase-associated lipocalin (NGAL), a ubiquitous 21–25 kDa iron-carrying protein of the lipocalin, and kidney injury molecule-1 (KIM-1) have been widely studied as markers of tubule health (8). Early release of NGAL and KIM-1 upon damage/injury to the renal tubules, including the proximal tubule, loop of Henle, and collecting ducts, has been reported (8, 9). Tubular injury markers, especially NGAL, were correlated with CKD incidence and progression (10). Glomerular damage and decline in its function are primarily reflected by an estimated glomerular filtration rate (eGFR) <60 ml/min/1.73 m^2^ and the presence of albuminuria.

The existing readouts of renal function decline, however, have limited ability to detect kidney disease at an early stage. Serum creatinine concentration, upon which eGFR calculations are based, increases only when approximately 40–50% of the kidney parenchyma is damaged (11). Furthermore, albuminuria can be absent in certain kidney diseases such as tubulointerstitial or hypertensive kidney diseases (12). Wilms tumor-1 (WT1) protein, a proven histologic biomarker of human podocytopathies, has the potential to diagnose early kidney disease (13–17). WT1 regulates the expression of various crucial genes, such as those required for maintaining podocyte architecture (16, 18, 19). The direct role of WT1 in controlling the transcriptional reprogramming of podocyte-expressing genes and its regulation in the tissue as a repair response to injury has been suggested (20). A loss of renal WT1 expression may initiate a catastrophic collapse of the entire podocyte-stabilizing system, leading to several glomerular diseases such as focal segmental glomerulosclerosis (FSGS) and diabetic nephropathy (DN) (15). Thus, WT1 could be an ideal candidate biomarker for early diagnosis as it is a transcription factor and a master gene regulator in podocytes (15, 21). However, kidney biopsy is highly invasive, making the follow-ups for disease progression or treatment response difficult. Detection of WT1 protein in the urine exosomes, reported by us and others, could be a promising non-invasive approach for the diagnosis of early kidney disease (13, 21–23). The aim of this study was to determine if WT1 protein in uE (uE-WT1) could be an early indication of kidney injury in CKD-naïve individuals. For this, uE-WT1 along with other tubular injury makers were estimated in CKD-naïve individuals without or with type 2 diabetes mellitus (T2DM) and/or hypertension (HTN). Acute kidney injury (AKI) is a known risk factor for chronic kidney disease (including damaged podocytes) and shares common pathophysiological conditions (24, 25). KIM-1 and NGAL are widely studied tubular injury markers in episodes of AKI (26). Nevertheless, there are studies to suggest the potential of KIM-1 and NGAL in chronic kidney disease prognosis, besides their potential as early kidney injury markers (27). Thus, we compared uE-WT1 and tubular injury markers in our study. We also determined changes in uE-WT1 protein levels and their relation to kidney injury in diabetic rats.

## Materials and methods

### Human study

The study was approved by the Institutional Ethics Committee of Sanjay Gandhi Postgraduate Institute of Medical Sciences (IEC Code 2018-139-EMP-106) and Pondicherry Institute of Medical Sciences (PIMS IEC No. RC 18/105). Participant recruitment and blood analysis were described previously (28). Briefly, individuals aged 18–60 years without or with diabetes and/or hypertension and reporting to have no CKD were enrolled after written informed consent. Fasting blood and second morning urine were collected.

### Biofluid analysis

Urine was pre-processed, and 10 ml was used to isolate exosomes. The exosomes were isolated using the ultracentrifugation method as described by us previously (7, 22, 29, 30) and stored at −80°C until further analysis. Exosome characterization was done using nanoparticle tracking analysis (NTA), flow cytometry, and immunoblotting. Urinary exosomal (uE) samples were used to estimate uE-WT1 by sandwich ELISA (EH1321, FineTest) as per the manufacturer's protocol. An aliquot of whole urine was used for the estimation of the following markers using solid-phase sandwich ELISA (Human Lipocalin-2/NGAL DuoSet kits, R&D Systems), albumin (DY1455), KIM-1 (DY1750B), and NGAL (DY1757). Other parameters, including urine creatinine, serum creatinine, triglycerides, cholesterol, and blood urea nitrogen (BUN), were estimated using an autoanalyzer (XL-640, Erba Mannheim, Germany). For the analysis, urine KIM-1, NGAL, and uE-WT1 were normalized with urine creatinine.

### Animal study

The Institutional Animal Ethics Committee (IAEC) of Sanjay Gandhi Postgraduate Institute of Medical Sciences (Registration No. 57/PO/ReBi/SL/99/CPCEA) approved the study. Fourteen male Wistar rats (*R. norvegicus*) weighing between 230 and 280 g and aged 90–100 days were obtained from the Indian Institute of Toxicology Research, Lucknow, India. The animals were acclimatized and housed as per our routine procedure (30). The standard chow diet and water were freely accessible to all the animals under study. Intraperitoneal administration of streptozotocin (STZ) (50 mg/kg body weight of rat) dissolved in 0.1 M citric acid buffer with pH 4.5 was used to induce diabetes after 18 h of fasting. Since there can be early mortality due to excessive insulin secretion by damaged beta cells, rats were provided with sucrose (15 g/L) supplemented with drinking water for 48 h. Diabetes in rats was confirmed at a blood glucose level of 11 mmol/L (1 mmol = 18 mg glucose). To monitor blood glucose using a glucometer (Optimum Exceed, Abbott Diabetes Care Inc., Alameda, CA, United States), blood from the tail vein was collected after 48 h of STZ injection. Urine samples were collected at baseline (before STZ injection) and at the 6th and 9th weeks after STZ-induced diabetes using metabolic cages (Lab Products, United States). Rats were euthanized, and the kidneys were harvested after the 9th week to confirm kidney injury by histopathology using periodic acid-Schiff (PAS) staining. Kidney tissue sections from vehicle-treated rats were used as controls.

### Nanoparticle tracking analysis

Nanoparticle tracking analysis (NTA using NanoSightNS300, Malvern Instruments, United Kingdom) was performed to analyze the size distribution and concentration of urinary exosomes. Briefly, exosomes were diluted in 1 × PBS and loaded into a sample chamber. A green laser was used to detect the nanoparticles, and five videos of 60 s duration were taken. Data were analyzed using the NTA 3.2 software (Malvern Instruments), which tracks the individual particles and calculates their size and velocity to generate a size distribution profile and determine the nanoparticle concentration.

### Histopathology

Kidney tissues were fixed in 4% paraformaldehyde. Thereafter, tissues were embedded in paraffin blocks and 3 μm slices were cut from embedded blocks for staining. Periodic acid-Schiff (PAS) staining was done following the manufacturer's protocol (Sigma, St. Louis, MO, United States) as described previously (29). Stained sections were visualized at 400 × by using an Olympus IX73 light microscope.

### Immunoblotting

For Western blotting of urinary exosomes, paired urine samples from rats collected at baseline (before STZ-injection), at the 6th and 9th weeks after STZ-injection, were used (*n* = 3 rats/time point). Western blotting of exosomal protein was performed as described by us previously (22). Briefly, exosomal protein samples were solubilized in Laemmli sample buffer. For each sample, an equal volume of solubilized protein was loaded onto a 10% polyacrylamide gel. Separated proteins were transferred onto nitrocellulose membranes and blocked with 5% non-fat dry milk for 1 h. Membranes were then incubated with a primary antibody against the WT1 protein (Abcam, MA, United States) overnight at 4°C. Thereafter, the incubation membranes were washed and then incubated with horseradish peroxidase-conjugated anti-rabbit secondary antibody (1:5,000). The antibody-antigen reactions were visualized using chemiluminescence (GE Healthcare, NJ, United States). Densitometry analysis of the uE-WT1 protein band was done. Total uE protein loaded on the gel was used for normalization.

For exosome characterization, a similar protocol was performed using antibodies against CD81 (Abcam, cat no. ab23505).

### Flow cytometry

Exosomes were analyzed for the presence of surface marker CD63 by flow cytometry using magnetic coated CD63-Dynabeads (Thermo Fisher Scientific, Waltham, MA, United States, Cat No. 10606D) as per the manufacturer's instructions. Isolated exosomes were resuspended in 1 × PBS and bound to magnetic-coated CD63-Dynabeads (Thermo Fisher Scientific, Waltham, MA, United States) overnight at 4°C. The following day, the Dynabeads-bound exosomes were incubated with an anti-CD63 mouse antibody (Abcam, Cat No. ab59479) overnight at 4°C along with an appropriate isotype control. After incubation, the beads were washed two times with 1 × PBS and stained with Alexa Flour 488 anti-mouse secondary antibody (detection antibody) for 1 h at 4°C and analyzed by flow cytometry (Beckman Coulter DxFLEX Flow Cytometer).

### Statistical analysis

Categorical variables were represented as frequency (percentage), while continuous variables were represented as median (interquartile range) or mean ± standard deviation. uE-WT1 was divided into four groups using the first, second, and third quartiles. The Kruskal-Wallis H test was used to test the significance of urinary and serum markers by ordering ACR categories and uE-WT1 quarters, followed by the Mann-Whitney U test with Bonferroni correction as the *post-hoc* test. Furthermore, Spearman correlation (rho) and stepwise multivariable linear regression were performed to test the linear association between variables and to determine which of the several urinary markers (uE-WT1, KIM-1, and NGAL) best determined the differences in ACR levels. The Spearman rank correlation was performed to test the correlation between variables. To compare the levels of ACR and uE-WT1 in rats before and after STZ injection (at the 9th week), a Wilcoxon signed rank test was used.

### Regression analysis

The model with age, gender, and BMI as the only explanatory variables was considered the base model. Gender was converted to a dummy variable as required for linear regression. A stepwise variable selection approach using multivariable linear regression was used to determine the performance of urinary markers in explaining variability in ACR. The influential points were identified through regression diagnostics, and sensitivity analysis was performed. The coefficient of determination (R^2^) and Akaike information criterion (AIC) were used to evaluate model performance.

A *p* < 0.05 was considered to be statistically significant. All analysis was performed using Stata 16 (StataCorp) and GraphPad Prism 8. The power of the study was calculated to be more than 80%.

## Results

### Exosome characterization using NTA, immunoblotting, and flow cytometry

**Figure 1:**
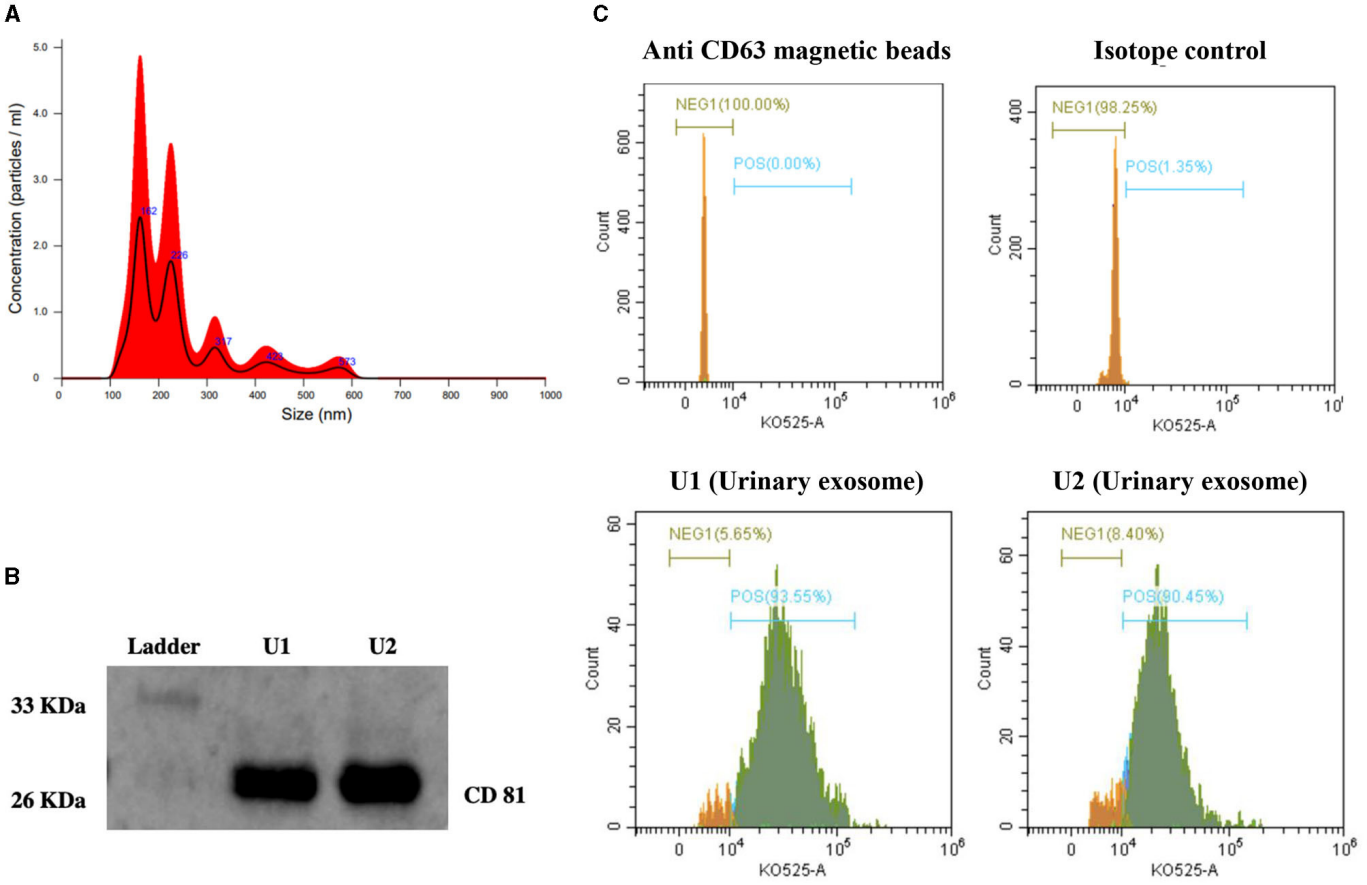
Characterization of exosomes from human urine. **(A)** Nanoparticle tracking analysis (NTA) showing exosome concentration (particles/mL)/size. **(B)** Representative immunoblot showing CD81 specific protein band in urinary exosomes samples (U1 and U2). **(C)** Histogram depicting the expression of CD63 on the surface of urinary exosome samples (U1 and U2) captured on anti-human CD63 magnetic beads using Flow cytometry analysis. (1) Unstained anti-human CD63 magnetic beads; (2) Anti-human CD63 magnetic beads with isotype control and stained with detection antibody; (3) and (4) Samples U1 and U2, captured on anti-human CD63 magnetic beads followed by anti-CD63 primary antibody and stained with detection antibody. Green color on histogram represents CD63 positive exosome population and orange color represents CD63 negative population.

NTA analysis showed that the maximum number of extracellular vesicles in our preparation had a particle size of 162 nm, with the second-highest concentration of 226 nm size (Figure 1A). Characterization by immunoblotting revealed a specific band for CD81 (exosome-specific marker protein) in our samples (Figure 1B). The percentage of exosome-bead complexes with positive staining for CD 63, another exosome-specific marker protein, was quantitated in our urinary exosome preparation using a flow cytometer. Figure 1C shows the results obtained in samples of the determined exosome-bead complex with 93.55% CD63 positive staining and 90.45% CD63 positive staining (Figure 1C).

### Individuals in the highest uE-WT1 quarter had elevated renal injury markers

We enrolled 627 participants reporting to have no CKD. However, 43 (6.86%) of them were found to have compromised kidney function (ACR≥30 mg/g or eGFR <60 ml/min/1.73 m^2^) after laboratory analysis. Individuals with kidney disease were removed from our study cohort; however, their characteristics were summarized in Supplementary Table 1. A total of 584 participants were involved in the study, including 312 (53.42%) men with an average [median (IQR)] age of 44.00 (36.00–51.00) years. In total, 335 (57.36%) of individuals had diabetes and/or hypertension (Table 1).

**Table 1:** Demographic, molecular, and biochemical characteristics of the study cohort.

**Variables**	**Total cohort (*n* = 584)**
Gender (male), *n* (%)	312 (53.42)
Age (years)	44.00 6.00–51.00)
BMI (kg/m^2^)	25.94 ± 4.84
Fasting blood glucose (mg/dl)	132.92 ± 61.55
Systolic blood pressure (mm of Hg)	129.67 ± 17.53
Diastolic blood pressure (mm of Hg)	82.52 ± 10.95
ACR (mg/g)	2.67 (1.29–5.03)
eGFR (ml/min/1.73 m^2^)	91.47 (77.65–102.78)
KIM-1 (ng/mg)	0.55 (0.23–1.28)
NGAL (ng/mg)	10.25 4.88–23.16)
uE-WT1 (ng/mg)	0.12 (0.06–0.21)
Triglyceride (mg/dl)	132.80 (97.00–181.25)
Cholesterol (mg/dl)	184.00 (153.00–217.00)
BUN (mg/dl)	10.75 (8.79–12.99)
Presence of diabetes and/or hypertension, *n* (%)	335 (57.36)
Duration of diabetes^*^ (years)	3 (1–7)

Values were reported as Mean ± SD, Median (IQR), or Frequency (%) wherever applicable.

KIM-1, kidney injury molecule-1; NGAL, neutrophil gelatinase-associated lipocalin; ACR, albumin-to-creatinine ratio; uE-WT1, urinary exosomal Wilms tumor protein-1; BMI, body mass index; eGFR, estimated glomerular filtration rate; BUN, blood urea nitrogen.

^*^among individuals with type 2 diabetes mellitus.

To further check the association of uE-WT1 with kidney injury markers, the individuals with normal kidney function were stratified by uE-WT1 quartiles. The individuals in the highest uE-WT1 quarter showed elevated urinary KIM1 and NGAL, relative to lower quarters (Tables 2A, B). There were fewer men than women in the highest quarter relative to the gender ratio in the lower quarter (*p* < 0.01). However, urinary NGAL remained significantly elevated in individuals in the highest uE-WT1 quarter, even after age and gender stratification. The median age of the individuals between the quarters ranged between 42 and 46 years. The urine ACR was also statistically different among the four groups; it was highest (but well below the diagnostic cutoff) in the upper quarter (Table 2A). The four groups had similar eGFR levels and lipid profiles (Table 2A).

**Table 2A:** Comparison of demographic, serum, and urinary markers between WT1 quarters.

	**Quarter 1 (*n =* 146)**	**Quarter 2 (*n =* 146)**	**Quarter 3 (*n =* 146)**	**Quarter 4 (*n =* 146)**	***p*-value**
uE-WT1 (ng/mg)	0.04 (0.03–0.05)	0.09 (0.08–0.10)	0.15 (0.14–0.18)	0.37 (0.27–0.54)	<0.01
Gender (male), *n* (%)	94 (64.38)	82 (56.16)	73 (50.00)	63 (43.15)	<0.01
Age (years)	42.0 (33.0–50.0)	43.0 (36.0–50.0)	45.0 (36.0–51.0)	46.0 (39.0–52.0)	0.03
BMI (kg/m^2^)	26.05 ± 4.94	26.52 ± 4.68	25.37 ± 4.75	25.81 ± 4.95	0.09
ACR (mg/g)	1.94 (0.93–3.39)	2.06 (1.07–4.20)	2.86 (1.91–4.72)	4.87 (2.19–8.67)	<0.01
Individuals with high-normal ACR, n (%)	03 (2.05)	06 (4.11)	07 (4.79)	30 (20.55)	<0.01
eGFR (ml/min/1.73 m^2^)	92.22 (80.13–103.58)	90.42 (76.64–101.67)	92.30 (78.97–101.51)	89.73 (76.58–103.44)	0.79
KIM-1 (ng/mg)	0.41 (0.20–0.85)	0.50 (0.23–1.09)	0.68 (0.26–1.25)	0.76 (0.30–2.29)	<0.01
NGAL (ng/mg)	4.63 (2.41–9.02)	9.05 (4.80–21.60)	12.37 (7.53–25.60)	21.44 (10.02–39.99)	<0.01
Presence of diabetes and/or hypertension, n (%)	84 (57.93)	84 (58.33)	86 (59.72)	81 (56.25)	0.95
Triglyceride (mg/dl)	132.50 (95.0–190.2)	141.90 (110.1–184.3)	134.45 (96.50–186.30)	125.05 (90.20–166.60)	0.09
Cholesterol (mg/dl)	176.50 (148.00–211.00)	184.00 (161.00–221.00)	186.50 (158.00–218.00)	182.00 (147.00–222.00)	0.20
BUN (mg/dl)	11.26 (8.88–13.64)	10.79 (9.02–13.41)	10.61 (8.83–12.90)	10.23 (8.04–11.82)	0.02

The Kruskal-Wallis H test was used to compare differences between quarters for variables measured on a continuous scale. The chi-square test was used to test for the association of WT1 quarters with categorical variables. A p-value of <0.05 was considered to be statistically significant.

Values were reported as mean ± SD, median (IQR), or frequency (%) wherever applicable.

KIM-1, kidney injury molecule-1; NGAL, neutrophil gelatinase-associated lipocalin; ACR, albumin-to-creatinine ratio; uE-WT1, urinary exosomal Wilms tumor protein-1; BMI, body mass index; eGFR, estimated glomerular filtration rate; BUN, blood urea nitrogen.

**Table 2B:** *Post-hoc* analysis of variables found statistically significant by uE-WT1 quarters when compared using the one-way ANOVA.

**Variables significant by one-way ANOVA for uE-WT1 quartile study**	***z*** **statistic (*****p*****-value)**					
	**Q1-Q2**	**Q1-Q3**	**Q1-Q4**	**Q2-Q3**	**Q2-Q4**	**Q3-Q4**
Age	−1.22 (0.22)	−1.72 (0.09)	−2.81 (<0.01)	−0.54 (0.59)	−1.78 (0.07)	−1.09 (0.27)
ACR	−1.68 (0.09)	−3.56 (<0.01)	−7.60 (<0.01)	−1.91 (0.06)	−6.48 (<0.01)	−4.69 (<0.01)
KIM-1	−1.10 (0.27)	−1.89 (0.06)	−3.16 (<0.01)	−0.87 (0.39)	−2.37 (0.02)	−1.61 (0.11)
NGAL	−5.13 (<0.01)	−7.76 (<0.01)	−9.24 (<0.01)	−2.73 (0.01)	−5.08 (<0.01)	−2.89 (<0.01)
BUN	0.11 (0.91)	1.16 (0.25)	3.48 (<0.01)	0.95 (0.34)	3.25 (<0.01)	2.38 (0.02)

Mann-Whitney U test with Bonferroni correction was used as the *post-hoc* test to one-way ANOVA. A p-value of <0.01 was considered to be statistically significant. Q1, Q2, Q3, and Q4 represent the first, second, third, and fourth quarters of uE-WT1, respectively.

KIM-1, kidney injury molecule-1; NGAL, neutrophil gelatinase-associated lipocalin; ACR, albumin-to-creatinine ratio; uE-WT1, urinary exosomal Wilms tumor protein-1; BMI, body mass index; BUN, blood urea nitrogen.

### Association of uE-WT1 and kidney injury markers with ACR

KIM-1, NGAL, and uE-WT1 showed a positive association with ACR. The level of renal injury markers in the ACR category (11–30 mg/g), a.k.a. high-normal albuminuria, had significantly higher levels of KIM-1, NGAL, and uE-WT1 when compared to those with ACR below 1 mg/g or 1–10 mg/g (Tables 3A, B).

**Table 3A:** Comparison of demographic, serum, and urinary markers between different albumin-to-creatinine ratio (ACR) categories.

**Variables**	** <1 (*n =* 119)**	**1–10 (*n =* 485)**	**11–30 (*n =* 62)**	***p*-value**
Age (years)	45.0 (38.0–52.0)	45.0 (36.0–52.0)	47.0 (40.0–51.0)	0.32
Male/female, n	68/51	241/244	39/23	0.07
BMI (kg/m^2^)	26.02 ± 4.91	25.98 ± 4.90	25.08 ± 4.11	0.40
eGFR (ml/min/1.73 m^2^)	88.26 (75.48–100.73)	88.61 (72.43–100.72)	78.80 (59.22–102.32)	0.13
Presence of diabetes and/or hypertension, n (%)	61 (51.26)	282 (58.14)	41 (66.13)	0.18
KIM-1 (ng/mg)	0.38 (0.19–0.82)^*^	0.57 (0.24–1.28)^*^	1.88 (0.55–4.50)	<0.01
NGAL (ng/mg)	5.44 (3.09–11.92)^*^	10.83 (5.45–23.16)^*^	21.41 (11.29–37.93)	<0.01
uE-WT1 (ng/mg)	0.08 (0.03–0.14)^*^	0.12 (0.07–0.23)^*^	0.40 (0.17–0.71)	<0.01
Triglyceride (mg/dl)	134.6 (99.4–191.2)	135.20 (99.00–182.60)	137.0 (107.9–227.0)	0.47
Cholesterol (mg/dl)	182.0 (154.0–224.0)	186.0 (154.00–223.00)	177.0 (146.0–215.0)	0.32
BUN (mg/dl)	10.96 (9.21–13.64)	10.88 (8.97–13.41)	12.36 (8.13–14.67)	0.41

Values were reported as mean ± SD, median (IQR), or frequency (%) wherever applicable.

^*^Statistical significance with respect to high-normal albuminuria (11–30 mg/g) for which a p <0.01 was considered to be statistically significant after Bonferroni correction.

KIM-1, kidney injury molecule-1; NGAL, neutrophil gelatinase-associated lipocalin; ACR, albumin-to-creatinine ratio; uE-WT1, urinary exosomal Wilms tumor protein-1; BMI, body mass index; BUN, blood urea nitrogen; eGFR, estimated glomerular filtration rate.

**Table 3B:** *Post-hoc* analysis of variables found statistically significant by ACR categories when compared using the one-way ANOVA.

**Variables significant by one-way ANOVA for ACR study**	**z statistic (** ***p*** **-value)**		
	**(1)–(2)**	**(1)–(3)**	**(2)–(3)**
KIM-1	−2.54 (0.01)	−5.04 (<0.01)	−4.45 (<0.01)
NGAL	−4.22 (<0.01)	−5.19 (<0.01)	−3.54 (<0.01)
uE-WT1	−4.72 (<0.01)	−6.92 (<0.01)	−5.97 (<0.01)

Mann-Whitney U test with Bonferroni correction was used as the *post-hoc* test to one-way ANOVA. A p <0.02 was considered to be statistically significant. (1), (2), and (3) represents ACR categories <1 mg/g, 1–10 mg/g, and 11–30 mg/g.

KIM-1, kidney injury molecule-1; NGAL, neutrophil gelatinase-associated lipocalin; ACR, albumin-to-creatinine ratio; uE-WT1, urinary exosomal Wilms tumor protein-1.

**Figure 2:**
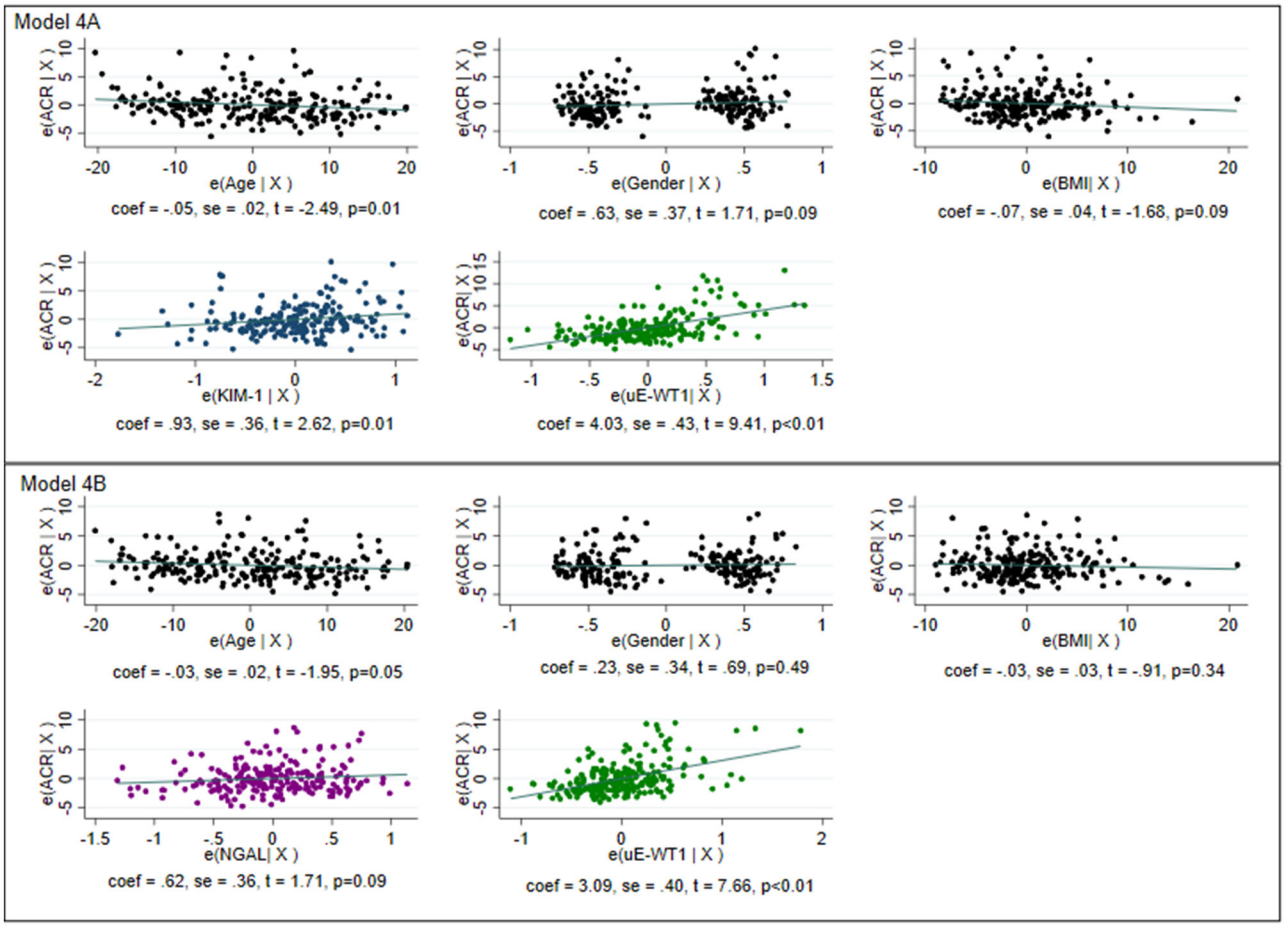
Graphical representation of multivariable linear regression of the model for determining variability within normoalbuminuria. The model was created after removing the influential points for individuals with normal albuminuria. The model with age, gender, and body mass index as the only predictors was considered the base model. *Model 4A*: Base model with KIM-1 and uE-WT1 as explanatory variables. The model could explain 35% of the variability in normoalbuminuria with an Akaike information criterion (AIC) of 1,067.23. *Model 4B*: Base model with NGAL and uE-WT1 as explanatory variables. The model could explain 28% of the variability in normoalbuminuria with an AIC of 1,031.68. Model 4B with a lower AIC is better than model 4B, where WT1 could independently determine high-normal albuminuria.

Correlation analysis showed a moderate but significant correlation between ACR and uE-WT1, (rho = 0.47 vs. rho = 0.25). Stepwise multivariable linear regression analysis was performed to determine the ability of uE-WT1, NGAL, and KIM-1 as determinants of high-normal ACR levels. The known urinary markers (KIM-1 and NGAL) were found to perform well in explaining the variability in ACR within the normal range (*p* < 0.01) (Supplementary Table 2). Base model with (1) KIM-1 could explain 5.9% of the variability in ACR, (2) NGAL could explain 8.70% of the variability in ACR, and (3) uE-WT1 could explain 24.50% of the variability in ACR. The performance of the regression model slightly improved after combining KIM-1 with uE-WT1 (${\text{R}}_{\text{adj}}^{2}$ = 0.273, AIC: 1,218.73) or NGAL with uE-WT1 (${\text{R}}_{\text{adj}}^{2}$ = 0.258, AIC: 1,237.26). After removing influential points, the model performance improved for uE-WT1 and KIM-1 (${\text{R}}_{\text{adj}}^{2}$ = 0.273 vs. 0.350, AIC = 1,218.73 vs. 1,067.23) as well as for NGAL and uE-WT1 (${\text{R}}_{\text{adj}}^{2}$ = 0.258 vs. 0.280, AIC = 1,237.26 vs. 1,031.68) (Figure 2, Supplementary Table 3).

### Early kidney injury in rats was associated with a rise in uE-WT1 protein levels

**Figure 3:**
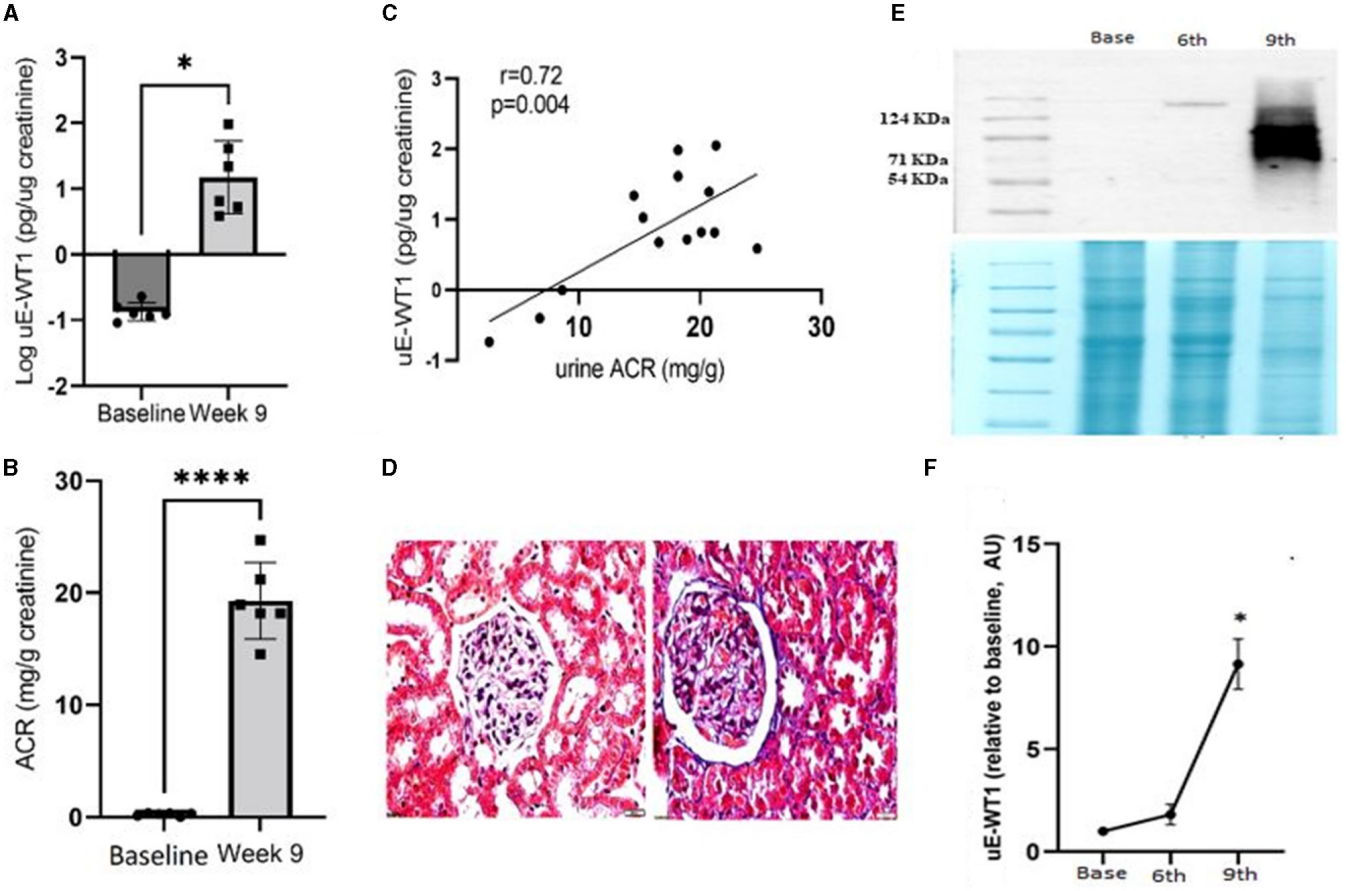
Association of uE-WT1 with kidney injury in rats. **(A)** Change in **(A)** WT1 protein levels in urinary exosomes (uE-WT1) and **(B)** urine albumin-to-creatinine ratio (ACR) in diabetic rats at the 9th week after STZ injection compared to their own baseline (before STZ-injection); *n* = 6 rats. **(C)** Scatter plot with regression fit line showing the relationship between uE-WT1 and ACR in diabetic rats at the 9th week after STZ injection (*n* = 14). **(D)** Representative image of periodic acid-Schiff (PAS) stained kidney tissue sections from diabetic rats (9th week after STZ injection) compared to age-matched vehicle-treated rats (*n* = 3/group). **(E)** Representative immunoblot showing the WT1 protein band in paired uE samples from rats before **(B)**, and at the 6th and 9th weeks of STZ-induced diabetes (*n* = 3 rats). Below is the coomassie-stained gel image showing the total protein loaded on the gel. **(F)** A line graph showing a summary of densitometry analysis of the immunoblots (*n* = 3/time point). uE-WT1 band density was normalized to total protein. ^*^p < 0.05 and ^****^p < 0.01.

We next tested if induction of kidney injury may cause a rise in uE-WT1 in diabetic rats. Wistar rats were made diabetic and then followed for 9 weeks to monitor diabetes-induced kidney injury. Rats were made diabetic by streptozotocin injections (i.p.). Analysis of uE-WT1 levels in the paired uE samples from these rats showed a significant rise at the 9th week of STZ injection, relative to baseline (Figure 3A). Furthermore, a substantial rise in uACR was also evident in these rats in the 9th week after STZ injections, relative to baseline (Figure 3B). Moreover, uACR strongly correlated with uE-WT1 protein levels in diabetic rats in the 9th week after STZ injection (Figure 3C). Histopathological changes were also confirmed in the kidney tissues by PAS staining in the 9th week after STZ injection (Figure 3D), similar to what has been reported by us previously (29). PAS staining clearly indicates considerable deposition of polysaccharides (collagen, glycogen) in rats after the 9th week of STZ injection compared to the kidney tissue section of age-matched vehicle-treated rats.

Moreover, the change in uE-WT1 protein levels was also confirmed by immunoblotting of the paired uE samples collected at baseline and the 6th and 9th weeks from diabetic rats (*n* = 3/time point). WT-1 protein bands were detected at the 6th and 9th weeks after STZ injection, while no band was detected in the baseline uE samples (Figure 3E). The densitometric analysis confirmed a significant rise in band density at 9th-week uE samples relative to baseline (Figures 3E, F).

## Discussion

The limited ability of the existing biomarkers to detect CKD at its earliest stage has led to the quest for novel biomarkers. Wilms tumor protein (WT1), a well-proven histological marker of podocytopathies in human renal biopsies, has the potential to detect early glomerular injury (31). However, kidney tissue biopsy for predicting/screening CKD is not feasible. Estimating WT1 protein concentration in uE could be a non-invasive alternative (22). Urinary loss of WT1 was reported in patients with FSGS, as indicated by its high expression in urine exosomes (22). Previously, we also reported the predominant presence of WT1 protein in uE from type 1 DM patients and its strong association with reduced renal function (22). However, the published reports on uE-WT1 analysis to date relied on semiquantitative immunoblotting (15, 22, 23, 32). In this study, we estimated WT1 protein concentrations by ELISA for the first time in uE from humans to determine its potential as an early indicator of kidney injury in CKD-naïve individuals. NTA, immunoblotting, and flow cytometry analysis confirm the presence of a urinary exosome population in our isolated samples.

Our study showed that participants in the highest uE-WT1 quarter had elevated tubular injury markers, NGAL and KIM-1. All the uE-WT1 quarters had a similar proportion of individuals with either diabetes and/or hypertension, known risk factors for CKD. However, there were more individuals with high-normal albuminuria in the highest uE-WT1 quarter. High-normal albuminuria is defined as high ACR levels below the diagnostic cutoff (ACR <30 mg/g). Although yet to be established, high-normal albuminuria has been reported as a risk for incident CKD in low-risk individuals (33). In fact, previous studies on CKD risk in the general population have defined optimal ACR levels as <1.13 mg/mmol and high-normal ACR as 1.13–3.40 mg/mmol (34). Besides, NGAL concentrations of 35.2 ng/ml and KIM1 174.95 pg/ml have been associated with tubule injury in humans (35). NGAL in transient AKI (1.7 ng/mg) or persistent AKI (8.9 ng/mg) was lower than what we found in individuals in the highest uE-WT1 quarter (36). Furthermore, NGAL and uE-WT-1 also showed a moderate but significant correlation with ACR in CKD-naïve individuals. We further found that the age, body mass index, and gender-adjusted model with uE-WT1 performed better than either NGAL or KIM-1 as determinants of high-normal albuminuria.

In one of our previous studies on type 1 DM individuals, the higher band density of WT1 in uE was associated with reduced renal function (22). Furthermore, a detectable WT1 protein band was seen in the uE of only 50% (15 out of 30) of the non-proteinuric type 1 DM patients in that study cohort, as opposed to 1 out of 24 healthy individuals (22). The absence of detectable bands in samples with lower uE-WT1 concentrations was anticipated by a less sensitive and qualitative approach. Nevertheless, the data further support the idea that a detectable band or elevated uE-WT1 could be an indication of early kidney injury in humans. Moreover, a detectable WT1 protein band was observed in the uE of FSGS animals before proteinuria or glomerular histological damage, supporting the early diagnostic ability of uE-WT1 (15). We also confirmed the rise in WT1 protein levels in the uE of rats after diabetes-induced kidney injury; the rise was strongly associated with uACR. Together, these findings suggest that elevated uE-WT1 concentrations could diagnose early CKD; however, longitudinal studies are warranted to test if uE-WT1 could also predict incident CKD.

The underlying mechanisms of the observed association between uE-WT1 changes and reduced kidney function could be in line with the known significance of WT1 in regulating podocyte phenotype. The podocyte is one of the distinct components of the glomerular filtration barrier and a critical determinant of kidney function. WT1, being an important transcription factor, can bring out remarkable changes in the podocyte phenotype with the onset of kidney disease. An initial rise in renal WT1 levels has been suggested as a protective response to early injury, while the absence of an optimal protective response leads to podocyte dysfunction and damage (31). The elevated WT1 in human uE may indicate urinary loss and reduced renal levels. An increased WT1 expression has been reported in the uE of FSGS patients (15). The causative role of reduced renal WT1 in podocyte dysfunction and glomerulosclerosis is well recognized (37). Thus, increased urinary loss of WT1 may lead to podocyte dysfunction and increased glomerular albumin leakage that induces the release of early tubule injury markers (38–41).

Our study has a few limitations. First, the fasting status of the participants was self-reported, and the duration of fasting may vary between the participants. Second, eGFR was calculated using serum creatinine, which may have confounding effects due to differences in muscle mass. Third, kidney function was evaluated based on a single reading of serum creatinine and ACR. Fourth, the average duration of hypertension reported by the participants was 4 years; however, it was unclear to many of the participants.

Nevertheless, this is the first report where WT1 protein concentrations have been estimated in urine exosomes in humans. We report for the first time that individuals with high uE-WT1 concentrations have elevated tubular injury markers. Besides, uE-WT1 outperformed urinary NGAL and KIM-1 as determinants of high-normal albuminuria in the general population. However, longitudinal studies are warranted to confirm whether uE-WT1 could predict early kidney injury.

## Data availability statement

The original contributions presented in the study are included in the article/Supplementary material, further inquiries can be directed to the corresponding author.

## Ethics statement

The studies involving humans were approved by the Institutional Ethics Committee, SGPGIMS, Lucknow, India, and the Institutional Ethics Committee of Pondicherry Institute of Medical Sciences, Puducherry, India. The studies were conducted in accordance with the local legislation and institutional requirements. The participants provided their written informed consent to participate in this study. The animal study was approved by Institutional Animal Ethics Committee, SGPGIMS, Lucknow, India.

## Author contributions

SK: conception of the study, data collection and analysis, and initial draft. ST: conception of the study, analysis, and critical revision of the manuscript. AP: coordinated in data collection and critically revised the manuscript. PM and DB: critically revised the manuscript. MS, RS and SA: carried out the molecular experiment. All authors approved the manuscript.
