# Supplementary material for: Association of Wilms tumor-1 protein in urinary exosomes with kidney injury: a population-based cross-sectional study
Source: Front Med (Lausanne). 2023 Sep 18;10:1220309. doi: 10.3389/fmed.2023.1220309 (PMC10545876; doi:10.3389/fmed.2023.1220309)
Supplement: Supplementary file 1 [file Table_1.docx]

**Supplementary Table 1:** Demographic, molecular and biochemical characteristics of individuals with compromised kidney function (ACR≥30mg/g or eGFR<60ml/min/1.73m^2^) identified after laboratory analysis

| **Variables** | **Compromised kidney function**  **(n=43)** |
| --- | --- |
| Gender (Male), n(%) | 25(58.14) |
| Age  (years) | 47.00  (40.00-53.00) |
| BMI(Kg/m^2^) | 25.60±4.60 |
| ACR  (mg/g) | 12.60  (2.68-36.65) |
| eGFR  (ml/min/1.73m^2^) | 57.27  (49.08-83.33) |
| KIM-1  (pg/ml) | 986.73  (404.19-3364.23) |
| NGAL  (pg/ml) | 14022.91  (7510.22-33590.11) |
| Triglyceride  (mg/dL) | 142.70  (107.60-194.00) |
| Cholesterol  (mg/dL) | 187.00  (151.00-232.00) |
| BUN  (mg/dL) | 13.13  (10.56-17.38) |
| Presence of diabetes and/or hypertension,n(%) | 25(58.14) |

Values were reported as Mean±SD, Median(IQR) or Frequency(%) wherever applicable

KIM-1:Kidney injury molecule-1, NGAL: Neutrophil gelatinase-associated lipocalin, ACR: Albumin-to-creatinine ratio, BMI: body mass index, ACR: albumin-to-creatinine ratio, eGFR: estimated glomerular filtration rate, BUN: blood urea nitrogen

**Supplementary Table 2:** Stepwise model selection by multivariable linear regression to determine variability in ACR for individuals with normoalbuminuria and without diabetes. ACR is the outcome variable. Base model included age, gender and BMI as the only explanatory variables. Log-transformed KIM-1, NGAL and WT1 was used as explanatory variables. p≤0.05 is considered statistically significant.

|  | **Explanatory variables** | **Adjusted**  **R^2^adj** | **AIC** | **VIF** | **p value**  **(F-test)** |
| --- | --- | --- | --- | --- | --- |
| Base model | Age, gender and BMI | <0.059 | 1375.53 | 1.04 | 0.66 |
| Model 1 | Base model + Log10(KIM-1) | 0.061 | 1276.17 | 1.04 | <0.01 |
| Model 2 | Base model + Log10(NGAL) | 0.089 | 1284.11 | 1.06 | <0.01 |
| Model 3 | Base model + Log10(uE-WT1) | 0.250 | 1305.13 | 1.07 | <0.01 |
| Model 4a | Model 1 + uE-WT1 | 0.273 | 1218.73 | 1.12 | <0.01 |
| Model 4b | Model 2 + uE-WT1 | 0.258 | 1237.26 | 1.17 | <0.01 |

ACR: albumin-to-creatinine ratio; KIM-1: kidney injury molecule-1; NGAL: neutrophil gelatinase-associated lipocalin; uE-WT1: urinary exosomal Wilm’s tumor 1 protein

**Supplementary Table 3**: Multivariable linear regression analysis of final selected model to determine variability in ACR for LR individuals with normal albuminuria. *Model 4A*: Base model with KIM-1 and uE-WT1 as explanatory variables. The model could explain 35% of variability in normoalbuminuria with an Akaike Information Criterion (AIC) of 1067.23. *Model 4B*: Base model with NGAL and uE-WT1 as explanatory variables. The model could explain 28% of variability in normoalbuminuria with an AIC of 1031.68. Model 4B with lower AIC is better than model 4B.

| **Model 4A** | **Coefficient** | **Standard error** | **95%**  **confidence interval** | **t statistic** | **p value** |
| --- | --- | --- | --- | --- | --- |
| Age | -0.05 | 0.02 | -0.09 to -001 | -2.49 | **0.01** |
| Gender | 0.63 | 0.37 | -0.10 to 1.35 | 1.71 | 0.09 |
| BMI | -0.07 | 0.04 | -0.14 to 0.01 | -1.68 | 0.09 |
| Log10 (KIM-1) | 0.93 | 0.36 | 0.23 to 1.63 | 2.62 | **0.01** |
| Log10(uE-WT1) | 4.03 | 0.43 | 3.19 to 4.88 | 9.41 | **<0.01** |
| Constant | 10.67 | 1.27 | 8.17 to 13.16 | 8.42 | **<0.01** |
| **Model 4B** |  |  |  |  |  |
| Age | -0.03 | 0.02 | -0.07 to 0.00 | -1.05 | 0.05 |
| Gender | 0.23 | 0.34 | -0.43 to 0.90 | 0.69 | 0.49 |
| BMI | -0.03 | 0.03 | -0.10 to 0.04 | -0.91 | 0.34 |
| Log10(NGAL) | 0.62 | 0.36 | -0.09 to 1.33 | 1.71 | 0.09 |
| Log10(uE-WT1) | 3.09 | 0.40 | 2.30 to 3.89 | 7.66 | **<0.01** |
| Constant | 7.47 | 1.23 | 5.04 to 9.90 | 6.07 | **<0.01** |

ACR: albumin-to-creatinine ratio; KIM-1: kidney injury molecule-1; NGAL: neutrophil gelatinase-associated lipocalin; WT1: urinary exosomal Wilm’s tumor 1 protein
